# Supplementary material for: The Colorimetric Detection of the Hydroxyl Radical
Source: Int J Mol Sci. 2023 Feb 19;24(4):4162. doi: 10.3390/ijms24044162 (PMC9964436; doi:10.3390/ijms24044162)

Supporting Information

For

The Colorimetric Detection of the Hydroxyl Radical

Yandong Ran, Mohammed Moursy, Robert Hider and Agostino Cilibrizzi*

Institute of Pharmaceutical Sciences, King's College London, SE1 9NH, United Kingdom

*agostino.cilibrizzi@kcl.ac.uk

Content: spectral data for NPBG.

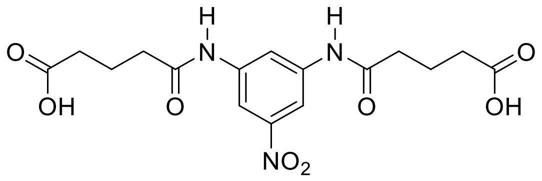

N,N'-(5-nitro-1,3-phenylene)bisglutoramide (**NPBG**)

$C_{16}H_{19}N_3O_8$
MW: 381.34

1. ^{1}H NMR – NPBG
2. ^{13}C NMR – NPBG
3. DEPT ^{13}C NMR – NPBG
4. MS (ESI$^-$) – NPBG
5. FT-IR – NPBG
6. UV spectra of the *ortho-* (a) and *para-* (b) hydroxy and phenolate NPBG species
7. HPLC chromatograms of *ortho-* and *para*-hydroxy-NPBG isomers monitored at 430 nm (a) and 232 nm (b)

1. ^{1}H NMR - NPBG

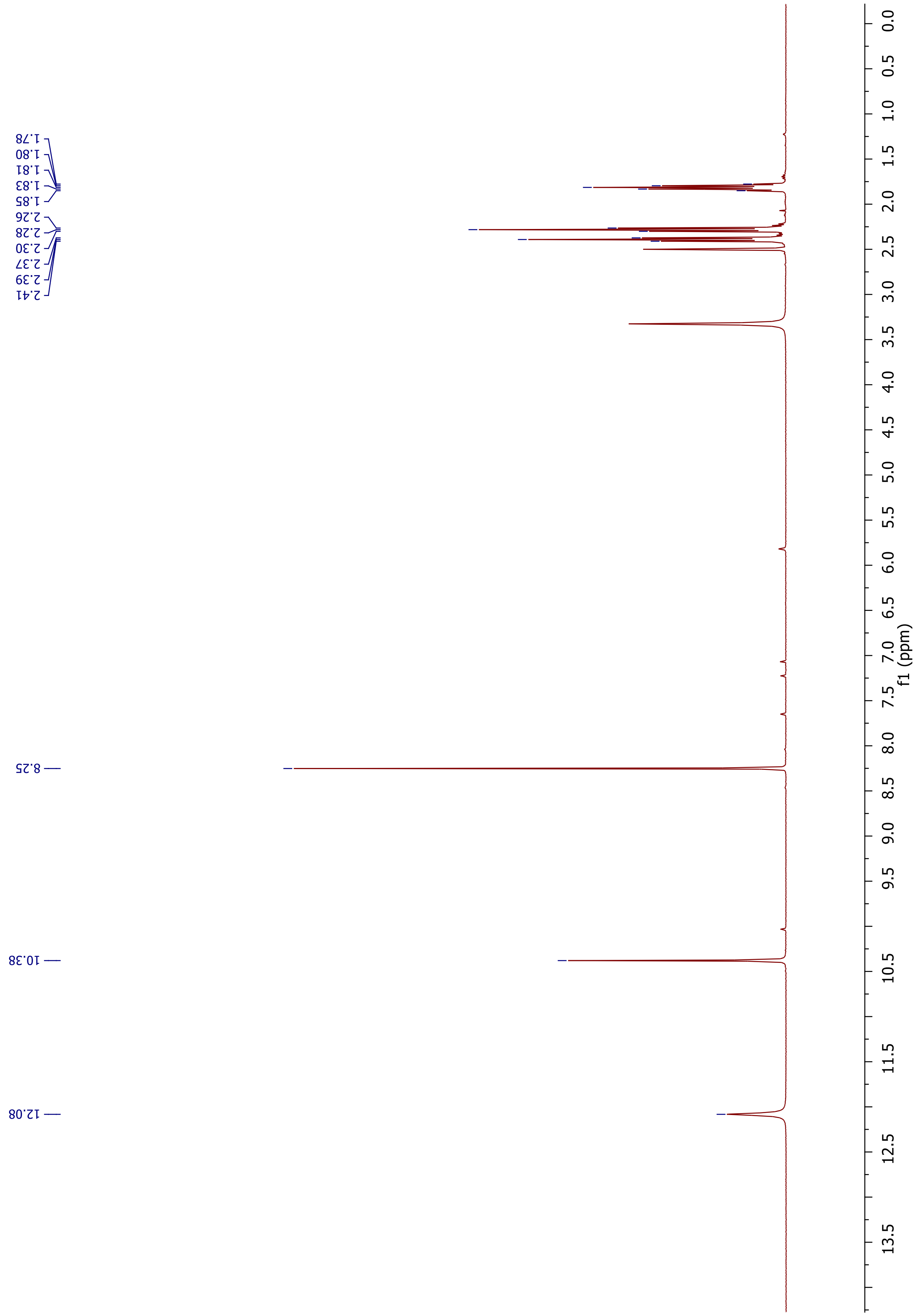

2. ^{13}C NMR – NPBG

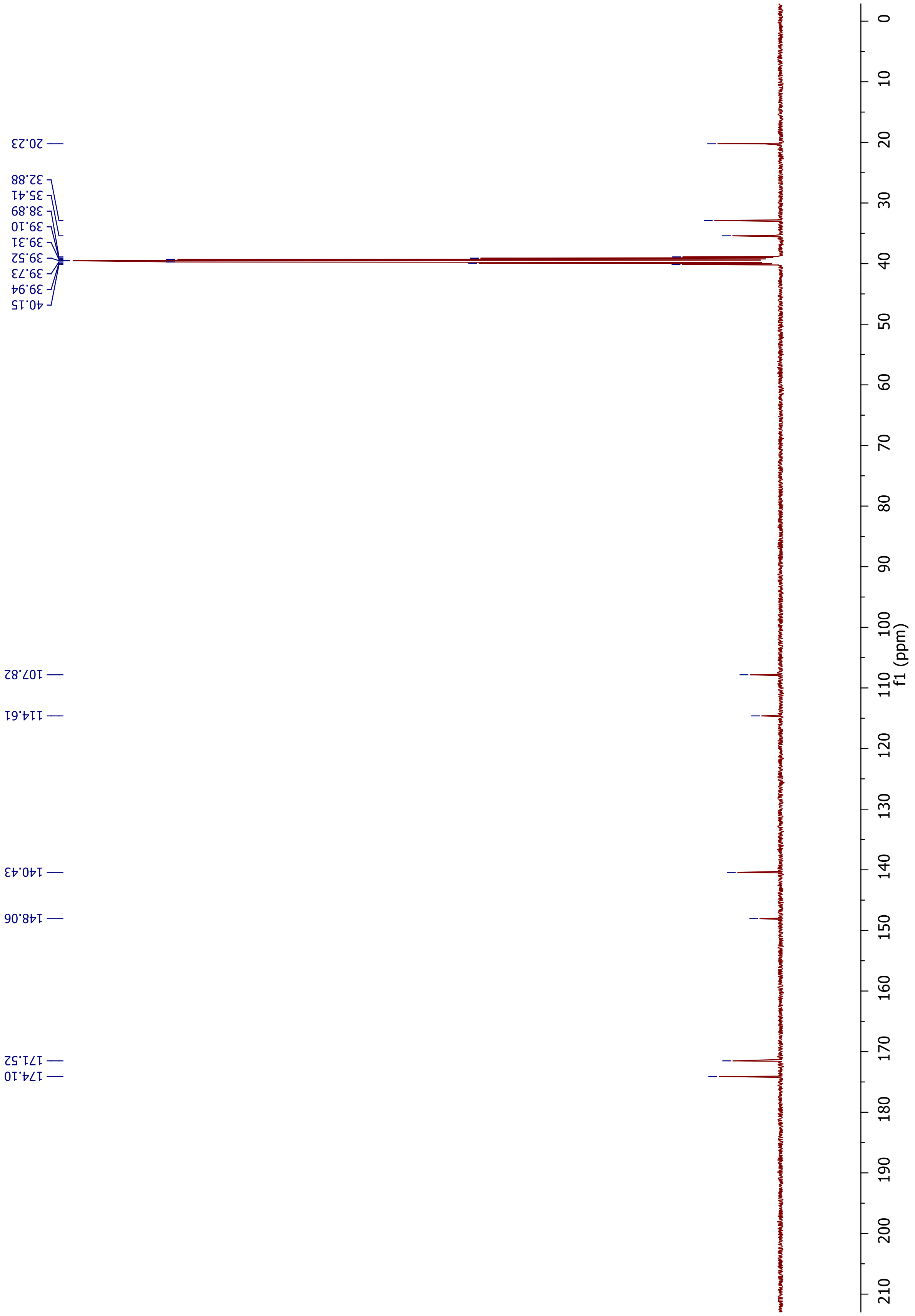

3. DEPT ^{13}C NMR - NPBG

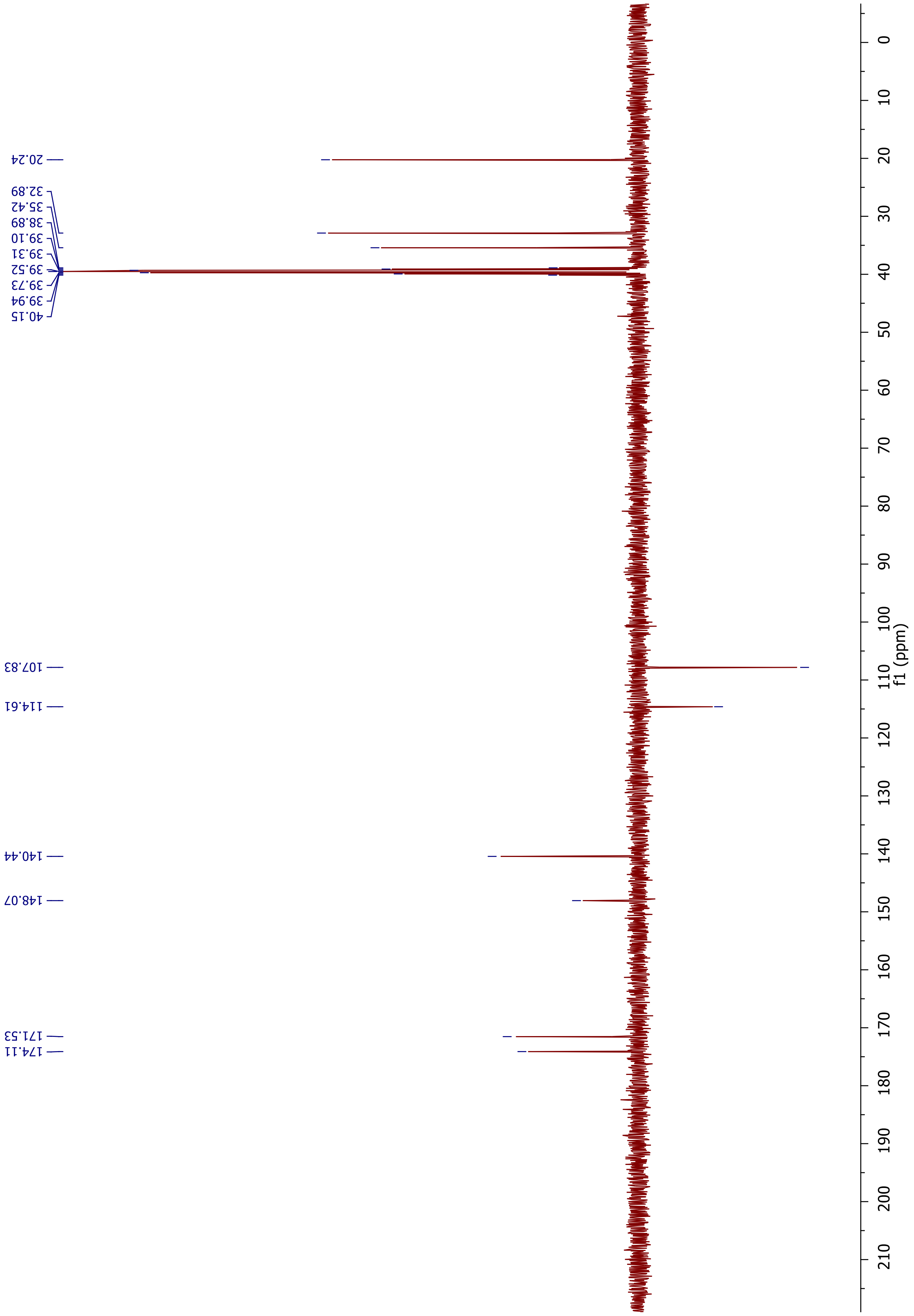

4. MS (ESI$^-$) - NPBG

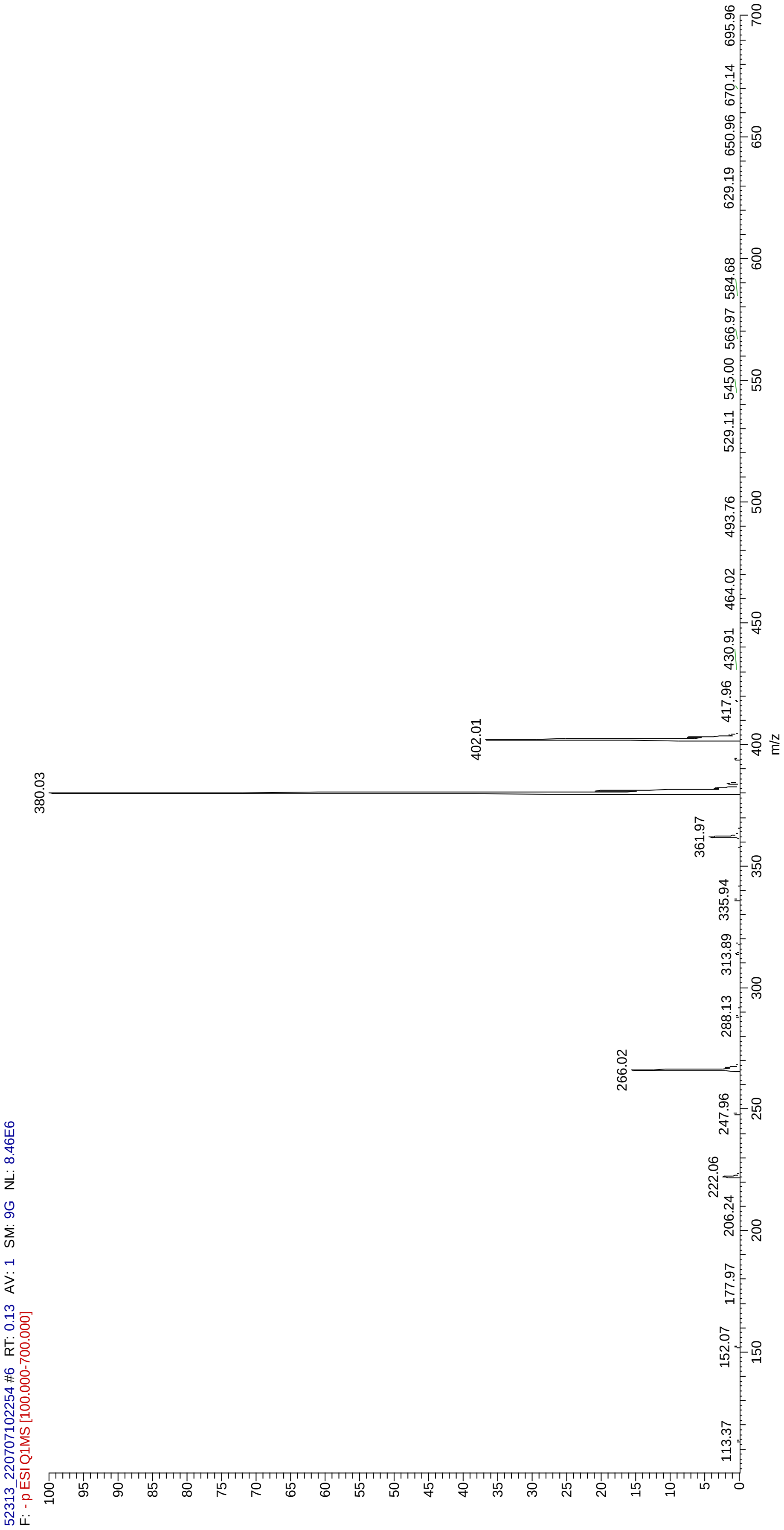
52313_22070710 2254 #6 RT: 0.13 AV: 1 SM: 9G NL: 8.46E6
F: - p ESI Q1MS [100.000-700.000]
380.03
402.01
266.02
361.97
113.37
152.07
177.97
206.24
222.06
247.96
288.13
313.89
335.94
417.96
430.91
464.02
493.76
529.11
545.00
566.97
584.68
629.19
650.96
670.14
695.96
m/z

5. FT-IR - NPBG

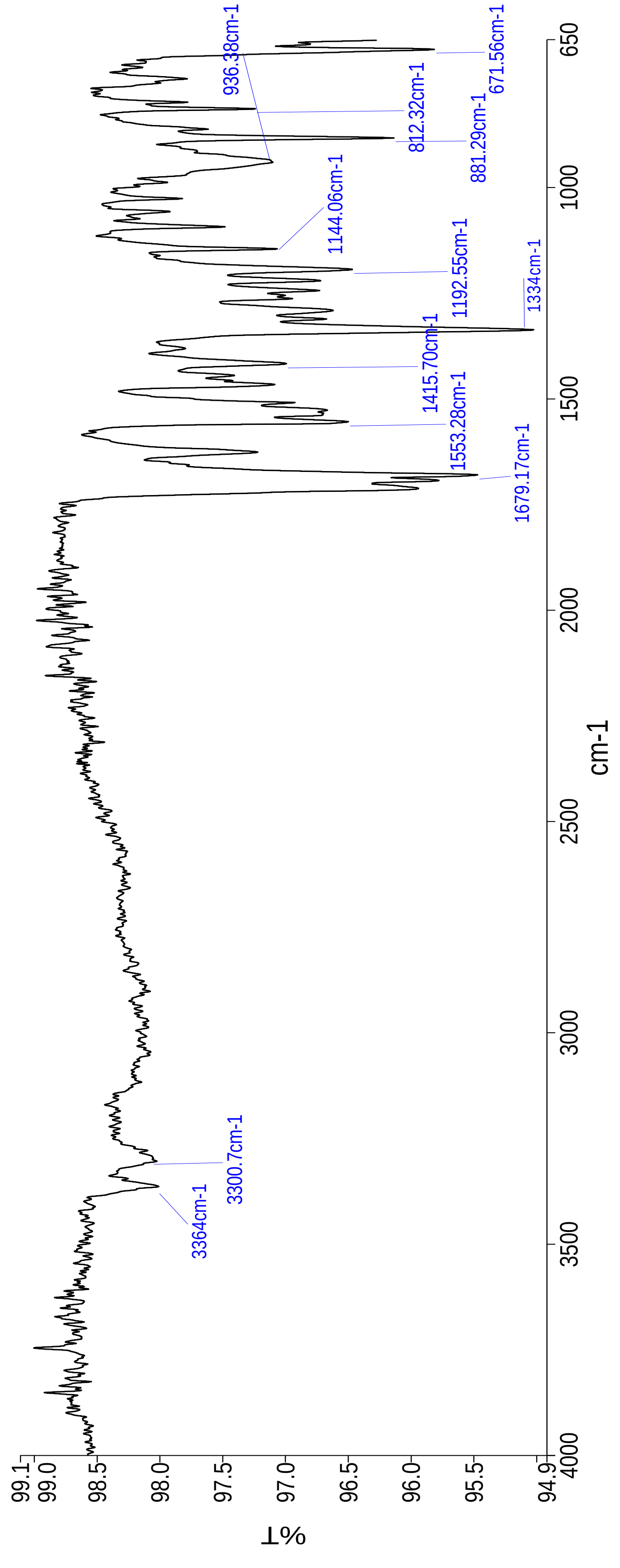

%T
cm-1
3364cm-1
3300.7cm-1
1679.17cm-1
1553.28cm-1
1415.70cm-1
1334cm-1
1192.55cm-1
1144.06cm-1
936.38cm-1
881.29cm-1
812.32cm-1
671.56cm-1

6. **UV spectra** of the *ortho*- (a) and *para*- (b) hydroxy and phenolate NPBG species

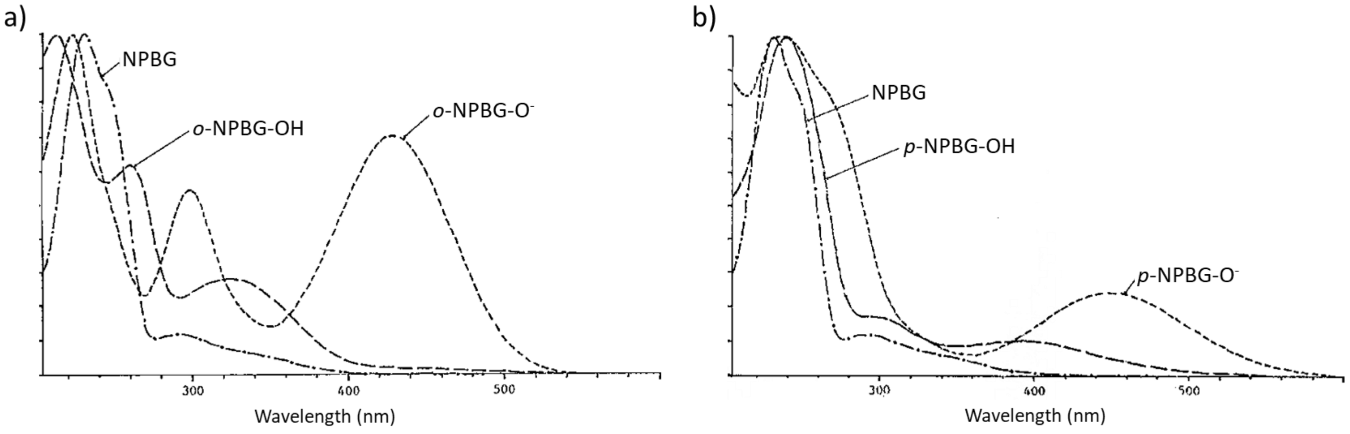

7. **HPLC chromatograms** of *ortho*- and *para*-hydroxy-NPBG isomers monitored at 430 nm (a) and 232 nm (b)

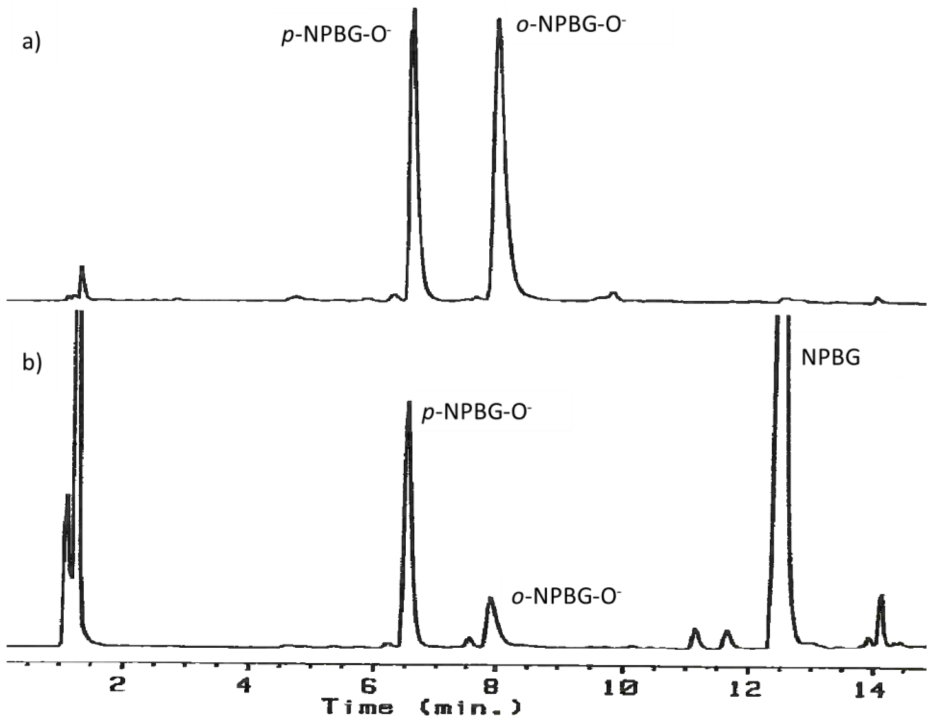

Supplement: Supplementary file 1 [file ijms-24-04162-s001.zip › ijms-2218684-supplementary.pdf]
